# A systematic review of economic evaluations for opioid misuse, cannabis and illicit drug use prevention

**DOI:** 10.1192/bjo.2023.515

**Published:** 2023-08-08

**Authors:** Jan Faller, Long Khanh-Dao Le, Mary Lou Chatterton, Joahna Kevin Perez, Oxana Chiotelis, Huong Ngoc Quynh Tran, Marufa Sultana, Natasha Hall, Yong Yi Lee, Cath Chapman, Nicola Newton, Tim Slade, Matt Sunderland, Maree Teesson, Cathrine Mihalopoulos

**Affiliations:** Monash University Health Economics Group, School of Public Health and Preventive Medicine, Monash University, Australia; Deakin Health Economics, Institute for Health Transformation, School of Health and Social Development, Deakin University, Australia; Monash University Health Economics Group, School of Public Health and Preventive Medicine, Monash University, Australia; School of Public Health, The University of Queensland, Australia; and Policy and Epidemiology Group, Queensland Centre for Mental Health Research, Wacol, Queensland, Australia; The Matilda Centre for Research in Mental Health and Substance Use, The University of Sydney, Australia; Monash University Health Economics Group, School of Public Health and Preventive Medicine, Monash University, Australia; and Deakin Health Economics, Institute for Health Transformation, School of Health and Social Development, Deakin University, Australia

**Keywords:** Illicit drug use, opioid, cannabis, economic evaluation, prevention

## Abstract

**Background:**

Substance use disorders negatively affect global disease burden. Effective preventive interventions are available, but whether they provide value for money is unclear.

**Aims:**

This review looks at the cost-effectiveness evidence of preventive interventions for cannabis use, opioid misuse and illicit drug use.

**Method:**

Literature search was undertaken in Medline, CINAHL, PsycINFO, EconLit through EBSCOhost and EMBASE, up to May 2021. Grey literature search was conducted as supplement. Studies included were full economic evaluations or return-on-investment (ROI) analyses for preventing opioid misuse, cannabis and illicit drug use. English-language restriction was used. Outcomes extracted were incremental cost-effectiveness ratios (ICER) or ROI ratios, with costs presented in 2019 United States dollars. Quality was assessed with the Drummond checklist.

**Results:**

Eleven full economic evaluation studies were identified from 5674 citations, with all studies conducted in high-income countries. Most aimed to prevent opioid misuse (*n* = 4), cannabis (*n* = 3) or illicit drug use (*n* = 5). Modelling was the predominant methodology (*n* = 7). Five evaluated school-based universal interventions targeting children and adolescents (aged <18 years). Five cost–benefit studies reported cost-savings. One cost-effectiveness and two cost–utility analysis studies supported the cost-effectiveness of interventions, as ICERs fell under prespecified value-for-money thresholds.

**Conclusions:**

There are limited economic evaluations of preventive interventions for opioid misuse, cannabis and illicit drug use. Family-based intervention (ParentCorps), school-based interventions (Social and Emotional Training and Project ALERT) and a doctor's programme to assess patient risk of misusing narcotics (‘the Network System to Prevent Doctor-Shopping for Narcotics’) show promising cost-effectiveness and warrant consideration.

Substance use disorders are a leading cause of global disease burden in terms of disability-adjusted life-years (DALYs).^1^ Drug use has been found to be associated with increased risk of unintended injuries, suicide, HIV infection, AIDS and liver cirrhosis, all of which contribute to greater disease burden.^1^ Moreover, there is evidence of substantial comorbidity between substance use disorders and other mental health disorders, which adds to the health burden associated with substance use disorders.^2,3^ In Australia, the annual economic costs including both healthcare costs and productivity loss associated with opioid misuse, cannabis and illicit drug use are substantial (over A$27 billion), and include cannabis (A$4.8 billion), opioids (A$17 billion) and methamphetamine (A$5.4 billion) in 2019 prices.^4–6^ Both health and economic burden highlight the substantial impacts of opioid misuse, cannabis and substance use disorders and the need to prevent them.

Economic evaluation is a tool used to determine whether a programme is cost-effective or good value for money, often providing critical information to assist decision makers in resource allocation and priority setting. A full economic evaluation compares costs and outcomes of two alternative interventions with results often presented as incremental cost-effectiveness ratios (ICERs).^7^ Cost–benefit analysis (CBA), cost-effectiveness analysis (CEA) and cost–utility analysis (CUA) are types of full economic evaluations. CEA outcomes are measured in natural or physical units such as a reduction in the risk of taking illicit drugs or delaying the initial use of a substance. CUA utilise a generic outcome of health gain that combines both quantity and quality-of-life components. Examples of generic outcomes include DALYs avoided or quality-adjusted life-years (QALYs) gained.^7^ Return-on-investment (ROI) studies are similar to CBA, wherein outcomes are measured in monetary values. However, ROIs usually only include cost-offsets and not health benefits.

There has been increasing global emphasis on adopting national strategies in the prevention of cannabis use, opioid misuse and illicit drug use. The UK and Australia, for example, have established national guidelines or strategies that highlight the importance of preventive interventions by focusing on reducing both the supply and demand.^8,9^ Interventions, such as school-based programmes and skills training, have been found to be effective in preventing or delaying substance uptake and preventing harms.^10,11^ Given this evidence, it is essential to know whether such interventions provide good value for money. Cost-effectiveness evidence has been adopted and is a requirement to support the funding and reimbursement of interventions and medications in the UK with the National Institute for Health and Care Excellence.^12^ Similarly, in Australia, although mental health interventions are not subjected to formal health technology assessment hurdles,^13^ implementation support from the Federal and State governments is provided after presenting a business case or a CBA where the benefits are presented in monetary values.^13,14^

There are two existing reviews published in 2021 that investigated economic evaluations for alcohol, smoking, illicit drug use prevention and other mental health disorders. The review by Nystrand et al,^15^ which identified a single economic evaluation for illicit drug use prevention, was limited to CUA studies and studies that have transferability to the Swedish setting. The second review published by Le et al^16^ included full economic evaluations for any mental health and substance use disorders, but did not have a specific focus on substances use prevention, as evident by the search strategy, which limits capture of relevant prevention evaluations. There are no published reviews of economic evaluations with a specific focus on preventive interventions for cannabis use, opioid misuse and illicit drug use or substance use disorders, leaving an important evidence gap for policy makers. Although cannabis is now legal in some jurisdictions, the legality of its use is still being debated in others; hence this review takes a broad approach in examining all preventive interventions.

## Objective

The objective of this review is to determine which interventions have ‘value for money’ evidence for the prevention of opioid misuse, cannabis and illicit drug use.

## Method

The current review follows the Preferred Reporting Items for Systematic Reviews and Meta-Analyses (PRISMA) guideline^17^ and was registered with the PROSPERO database (identifier CRD42020147386; the protocol was amended to include additional researchers and choose the Drummond checklist as the tool for quality assessment). This review is part of a broader project that includes a review of the cost-effectiveness of treatment interventions for substance use disorders (PROSPERO identifier CRD42020147403). During the course of the review, deviations from the registered protocol were warranted and decided upon. Initially, the review was planned to encompass all substances, including alcohol and tobacco prevention, but the number of eligible publications necessitated a decision to organise separate reviews for alcohol, tobacco and other substances. We also decided to present results of this particular review by specific substance (i.e. opioid misuse, cannabis and illicit drug use) to better illustrate which interventions have been evaluated for the prevention of its use, and to better reflect the variation in prevention approaches for these substances. The last protocol deviation was the use of the Drummond checklist for quality assessment in place of the Quality of Health Economic Studies Instrument (QHES). Although the QHES is appropriate to assess quality of modelled economic evaluations, the Drummond checklist is useful for both modelled and trial economic evaluations.

### Literature search

Electronic databases were searched for economic evaluations for alcohol, smoking, illicit drug use prevention and treatment in August 2019, and updated in May 2021. Electronic database searches were conducted in EMBASE and through the EBSCOhost platform for Medline, CINAHL, PsycINFO and EconLit databases. Further details of search terms are presented in Supplementary Appendix 1 available at https://doi.org/10.1192/bjo.2023.515. Search terms related to prevention and treatment, as well as terms for alcohol and smoking, opioid misuse, cannabis and illicit drug and substance use disorders, were included in the search. Treatment was explicitly included to expand the search because the terms ‘early interventions’, ‘early treatment’ and ‘preventive interventions’ are often used interchangeably. However, during the updated search for prevention, we decided to exclude the terminology ‘treatment’ because we identified no additional benefit of using this term during the initial screening. For this review, only cost-effectiveness studies that evaluated preventive interventions for opioid misuse, cannabis and illicit drug use (including problem use of prescription opioids) were included. Search strategies for specific databases differ on the subject headings used for their respective indexing. Subject headings were searched and uniquely utilised for each database except for EconLit. Keywords were used over subject headings in EconLit as subject headings were inappropriate. Other relevant free text search terms for titles and abstracts were first tested in Medline and were used in other databases through EBSCOhost. Filters were used to limit the searched articles to studies comprising peer-reviewed journal articles in English. No year restrictions were utilised. Grey literature search from cost-effectiveness registries was also conducted to supplement the search strategy.

### Inclusion and exclusion criteria

Studies were included if they were full economic evaluations (i.e. comparing at least two interventions including costs and outcomes) or ROI studies that evaluated a preventive intervention for cannabis use, opioid misuse or illicit drug use or use disorders, and were published in English. Cost-of-illness studies, partial economic evaluations (particularly cost–outcome description studies or cost analysis), book chapters and thesis papers were not eligible. Classification for prevention studies was primarily based on the study population (e.g. not diagnosed to have a substance use disorder or prevention of progressing to a substance use diagnosis) following the framework set by Mrazek and Haggerty.^18^ Specifically, studies examining interventions that prevent exposures or alter behaviours that can lead to opioid misuse, cannabis and illicit drug use. Our review includes preventive interventions both at a population level (such as policy legislation) and an individual level (such as educational programmes). Targeted prevention (e.g. targeting at-risk populations for opioid misuse, cannabis and illicit drug use) is also included in this review.

### Study selection and extraction

Articles retrieved from the online search were uploaded into Covidence (Veritas Health Innovation, Melbourne, Australia; see www.covidence.org), where duplicates were removed by the program. Two reviewers screened each article independently for inclusion during both title and abstract screening and full-text review stages (J.F., L.K.-D.L., M.L.C., J.K.P., O.C., H.N.Q.T., M. Sultana or N.H.). In the event of disagreement across the screening process, a third reviewer resolved the conflict. Data extraction commenced in November 2020 and was conducted by a single reviewer (J.F. or J.K.P.) in Microsoft Excel version 15.0 for Windows. The accuracy of extracted data was confirmed by a second reviewer (J.F. or J.K.P.). The results were extracted and synthesised in a descriptive and tabular format, which included information on the type of study, perspective and time horizon, costs and outcomes, and ICERs. Costs and ICERs were converted into 2019 United States dollars (US$) with Cost Converter version 1.6 (see https://eppi.ioe.ac.uk/costconversion/default.aspx) developed by the Co-convenors of the Campbell & Cochrane Economics Methods Group and Evidence for Policy and Practice Information and Co-ordinating Centre, for comparison across studies.^19^ For studies not reporting a reference year for costs, the assumption of 2 years before publication was used as the base year. Meta-analysis was not attempted because of the expected methodological heterogeneity across studies. Studies were grouped as prescription opioid misuse, cannabis use and illicit drug use prevention to account for cannabis being legal in some jurisdictions.

### Quality assessment

The Drummond ten-point checklist for assessing economic evaluations was used to evaluate the quality of included studies.^7^ The ten points for assessment include the research question, intervention(s) description, intervention effectiveness, costing methodology (identification, measurement and valuation), discounting or time preference, incremental analysis, sensitivity and uncertainty analysis, and presentation and discussion of results. The ten points include 33 sub-questions individually answered during quality assessment by two independent assessors (J.F., L.K.-D.L., M.L.C., J.K.P., O.C., H.N.Q.T., M. Sultana or N.H.). Conflicts were initially resolved by the quality assessors, with further unresolved conflicts adjudicated by a senior researcher (L.K.-D.L. or M.L.C.). Quality assessment identified which aspects highlighted by the Drummond checklist were met. A previously published scoring system was used to attach a score for each study.^20^ Each criteria could be given a score of 1 for a ‘yes’, 0.5 for a ‘cannot tell’ or 0 for a ‘no’. A quality score of 9 to 10 is considered ‘good’, a score of 6 to 8.5 is considered ‘fair’ and a score ≤5.5 is considered poor quality.

## Results

### Study selection and inclusion

**Fig. 1:**
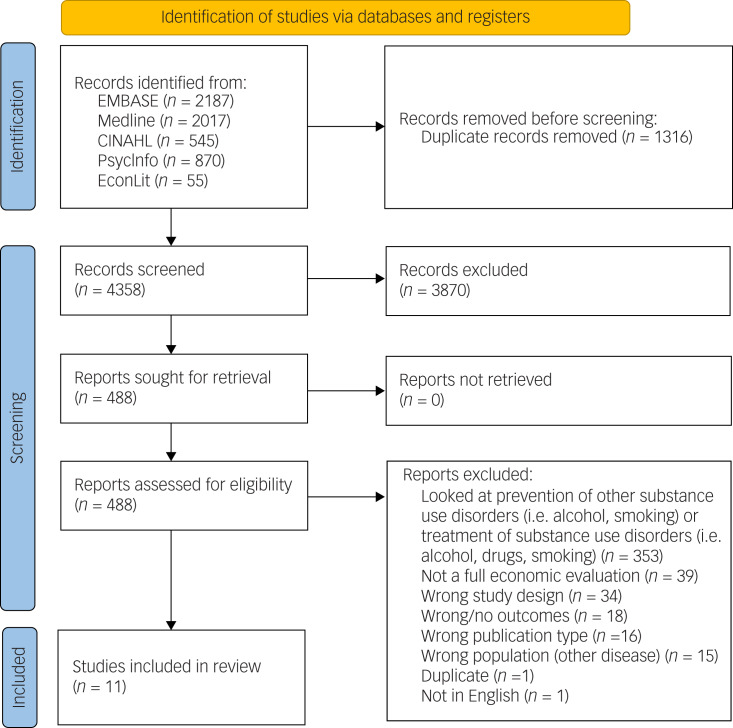
Preferred Reporting Items for Systematic Reviews and Meta-Analyses (PRISMA) diagram.

The literature search identified 5674 articles. After removing duplicates and screening the abstracts, 488 articles remained and were assessed in full-text review, resulting in 124 studies with further exclusions. The main reasons for exclusion are presented with the PRISMA diagram, with the most common reason being incorrect study design or not a full economic evaluation. A total of 364 prevention and treatment articles were identified that met the inclusion criteria. Of these, 11 articles dealt with cannabis use, opioid misuse and illicit drug use prevention, and met the full inclusion criteria for this review (Fig. 1).

### Study characteristics

As shown in Table 1, problem prescription opioid use (*n* = 4) and cannabis use (*n* = 3) prevention were evaluated in the majority of included studies, whereas other illicit drug use prevention was evaluated in two studies (cocaine, ecstasy and methamphetamine). Two studies considered non-specified illicit drug use prevention. Nine studies used a single type of economic analysis (three studies each for CBA, CEA and CUA). Multiple types of evaluation frameworks (e.g. CUA/CBA and CEA/CBA) were used in combination for two studies. In terms of study methods, economic modelling was utilised in the majority of studies (*n* = 7), followed by pre–post study design (*n* = 3) and a randomised controlled trial (RCT) incorporating economic modelling. The majority of the studies (*n* = 7) were conducted in the USA, followed by Sweden (*n* = 2). The UK and South Korea had one study each. Children (aged 5–14 years) were the main target population in 70% of the studies, which involved school-based and family-centred (universal) interventions. Adults were the target population in the remaining studies, evaluating selective preventive interventions.

**Table 1:** Study details

Lead author (year), country	Substance use disorder studied (smoking, alcohol, drugs or any combination)	Population description (universal, selective, indicated)	Intervention(s) and comparator (setting, individual or group-based, parenting)	Evaluation type	Study design (trial (*N*) or modelled, pre–post study)	Perspective, time horizon, year of pricing, discount rates, currency	Cost categories	Outcomes	Results, ICERs, sensitivity analysis (key points) (US$ 2019 equivalent)
Crowley et al (2014),^21^ USA	Prescription opioid	Sixth grade students and their families (universal)	Intervention(s): (1) SFP:10–14 only (2) All Stars programme only (3) LST only (4) Project ALERT only (5) SFP:10–14 + All Stars (6) SFP:10–14 + LST (7) SFP:10–14 + Project ALERT (8) No programme only (comparator)	CEA	Modelling (decision tree - propensity and marginal structural model)	Perspective: none Time horizon: 6 years Reference year: 2010 Discount rate: 3% Currency: US$	Intervention costs	Incremental cost of preventing a youth from ever misusing prescription opioids before 12th grade	Base-case analysis: under WTP: $7500 ($8667) LST only: $613 ($717) to prevent one youth from misusing prescription opioids before 12th grade SFP:10–14 + LST: $3959 ($4628) to prevent one youth from misusing prescription opioids before 12th grade SFP:10–14 + All Stars: $4923 ($5755) to prevent one youth from misusing prescription opioids before 12th grade
Deogan et al (2015),^22^ Sweden	Cannabis	Eighth graders (universal)	Intervention(s): (1) Project ALERT (2) ordinary alcohol, tobacco, and other drug education equivalent (comparator)	CUA	Modelling (Markov)	Perspective: societal Time horizon: 20 years Reference year: 2013 Discount rate: 3% Currency: Euro	Intervention costs, cannabis consumption costs, healthcare (in-patient and out-patient) costs, municipality or government costs, legal and criminal justice system costs, productivity costs	Incremental cost per QALY gained	Base-case analysis: €22 384 ($2845) per QALY gained (20-year time horizon), cost-effective at €50 000 ($6356) per QALY WTP threshold Sensitivity analysis: €926 709 ($117 801) per QALY gained for 10-year time horizon, €14 981 ($1904) per QALY gained for lifetime time horizon
Guyll et al (2011),^23^ USA	Methamphetamine	Sixth and seventh graders (universal)	Intervention(s): (1) ISFP (2) LST (3) LST + SFP:10–14 (4) Minimal contact (comparator)	CEA, CBA	Economic evaluation alongside RCT and modelling	Perspective: employer Time horizon: trial: 5.5–6.5 years (CEA); model: 18–65 years or career time horizon (CBA) Reference year: 2006 Discount rate: 3% Currency: US$	Intervention costs, productivity costs, employer-funded healthcare costs	Cost per PYMU prevented Benefit–cost ratio Net benefit	Base-case analysis: ISFP versus control: $25 385 ($31 664) per PYMU prevented; 3.84 benefit for each $1; $2814 ($3510) net benefit LST versus control: $5112 ($6377) per PYMU prevented; 19.04 benefit for each $1; $2273 ($2835) net benefit LST + SFP:10-14 versus control: $62 697 ($78 206) per PYMU prevented; 1.56 benefit for each $1; $620 ($773) net benefit
Hajizadeh et al (2017),^24^ USA	Drug misuse (general)	Pre-kindergarten children (universal)	Intervention(s): (1) ParentCorps (family-centred enhancement to pre-kindergarten programming) (2) Standard pre-kindergarten programming (comparator)	CBA, CUA	Modelling (Markov)	Perspective: societal Time horizon: lifetime Reference year: 2015 Discount rate: 5% Currency: US$	Intervention, healthcare (drug misuse, obesity, and diabetes), judiciary costs and employment	Costs from development of obesity, diabetes and drug misuse health states including sequelae QALYs gained	Base-case analysis: savings of $4387 ($4703) per person in healthcare, criminal justice and productivity expenditures Increased individual's quality-adjusted life expectancy by 0.27 QALYs
Kim et al (2021),^25^ South Korea	Opioid	Patients prescribed opioids (selective)	Intervention(s): (1) Opioid misuse prevention programme embedded in the Narcotics Information Management System (‘the Network System to Prevent Doctor-Shopping for Narcotics’) (2) No programme (comparator)	CUA	Model (Markov)	Perspective: Korean healthcare perspective (base) and societal (sensitivity) Time horizon: 30 years Reference year: 2019, Discount rate: 5%, Currency: US$	Costs of the prevention programme, opioids and overdoses Transportation costs and productivity loss included for sensitivity analysis	QALYs	Incremental cost: $1.15 Incremental QALY: 0.00505 ICER: $227.26 per QALY Sensitivity: dominant because of savings in the transportation costs of the misuse state and productivity loss from premature death
Klapp et al (2017),^26^ Sweden	Drug misuse (general)	Seventh to ninth grade students (universal)	Intervention(s): (1) Social and emotional learning intervention - SET (2) No SET (comparator)	CBA	Pre–post test	Perspective: societal Time horizon: 5 years Reference year: 2013 Discount rate: 3.5% Currency: US$	Intervention costs, costs of drug utilisation to society	Intervention costs Monetized benefits (drug use costs to society)	Base-case analysis: Cost-benefit ratio: 13.9 per dollar spent Intervention cost: $540 ($596) Intervention benefits: $7510 ($8292) NPV: $6970 ($7695) Sensitivity analysis (a) Smaller cohort: 6.6 per dollar spent; $6374 ($7037) NPV (b) Heavy users only: 7.0 per dollar spent; $3220 ($3555) NPV (c) 60% fade-out after first year: 5.6 per dollar spent; $2460 ($2716) NPV
Kumar et al (2019),^27^ USA	Prescription opioid	Non-cancer, chronic pain patients (adults) (selective)	Intervention(s): (1) Misuse-deterrent formulation opioids (2) Non-misuse-deterrent formulation, extended-release opioids (comparator)	CEA	Modelling (epidemiological cohort)	Perspective: healthcare sector Time horizon: 5 years Reference year: 2017 Discount rate: none Currency: US$	Prescription costs, healthcare costs (including non-opioid-related costs and overdose costs)	Healthcare costs, new cases of misuse, person-years of misuse and opioid-related overdose	Base-case analysis: $232 000 ($241 450) per new misuse case prevented $81 000 ($84 299) per misuse years prevented $1.3 billion ($1.35 billion) per opioid overdose death prevented Sensitivity analysis: Cost neutrality: requires 40% reduction in misuse-deterrent formulation prices or 43% risk reduction
Mitchel et al (1984),^28^ USA	Alcohol, cannabis and smoking	Ninth grade students (universal)	Intervention(s): (1) Educational-type programme on prevention integrated in curriculum of health (2) Educational-type programme on prevention integrated in curriculum of religion (3) Educational-type programme on prevention integrated in curriculum of social studies (4) Non-exposed (comparator)	CEA	Pre–post study (*N* = 250)	Perspective: none Time horizon: 10 months Reference year: 1980 Discount rate: none Currency: US$	Programme development and provision costs	Cost per reduction in consumption of cigarettes, beer, wine, liquor, marijuana, non-prescription drugs, inhalants	Base-case analysis: $68 ($181) per student reduction in marijuana and cigarette consumption (programme cost $10 188 ($26 890) per 150 religion course students )
Paltzer et al (2019),^29^ USA	Alcohol, drugs and smoking	Adults (18–64 years) (universal)	Intervention(s): (1) SBIRT (2) No SBIRT (comparator)	CBA	Pre–post study (differences-in-differences design)	Perspective: healthcare Time horizon: 2 years Reference year: 2018 Discount rate: none Currency: US$	Intervention cost; healthcare costs	Changes in out-patient and in-patient days, in-patient admissions, emergency department admissions and corresponding costs or saving associated	Base-case analysis: Intervention cost: $51.05 ($52) Annual cost savings: $833.01 ($848) Total net cost savings: $781.87 ($796)
Pennington et al (2018),^30^ UK	Drug use (cannabis, ecstasy, cocaine)	Cannabis Study 1: Children whose parent use drugs (selective) Study 2: Occasional drug users (indicated) Study 3: Children and young people who are in contact with young offender teams, but not in secure environments (selective) Ecstasy Study 4: Occasional drug users (indicated) Cocaine Study 5: Young gay and bisexual men (selective) Study 6: Men who have sex with men (selective) Study 7: People considered homeless (selective)	Intervention(s): Cannabis use: (1a) Focus on Families (1b) Standard methadone treatment (comparator) (2a) Web-based feedback intervention (2b) Assessment only (comparator) (3a) Familias Unidas (3b) Community practice (comparator) Ecstasy use: (4a) Single session motivational and cognitive–behavioural intervention (4b) Assessment only (comparator) Cocaine use: (5a) Motivational interviewing in young gay and bisexual men (5b) Educational videos and structured discussion (comparator) (6a) Motivational interviewing among men who have sex with men (6b) Educational videos (comparator) (7a) STRIVE family intervention among newly homeless youth (7b) Standard care (comparator)	CUA	Modelling (decision tree) (1) Cannabis use model (2) Ecstasy use model (3) Cocaine use model	Perspective: partial societal Time horizon: Cannabis model: Study 1: 12 months; Study 2-3: 24 months Ecstasy model: Study 4: 12 months Cocaine model: Study 5-7: 24 months Reference year: 2015 Discount rate: 3.5% Currency: £	Cannabis model: Intervention cost, crime costs, psychosis related healthcare costs Ecstasy model: Intervention cost, crime costs, emergency department and hospital admission costs, mortality cost, drug dependence treatment costs Cocaine model: Intervention cost, crime costs, cocaine-specific hospital admission costs, cocaine-related cardiovascular hospital admissions, drug dependence treatment costs	Cost per QALY gained	Base-case analysis: None of the interventions were cost-effective at £20 000 ($31 014) per QALY Cannabis use: Study 1: £99 254 920 ($153 915 305) per QALY gained Study 2: £478 296 ($741 697) per QALY gained Study 3: £240 994 ($373 711) per QALY gained Ecstasy use: Study 4: £471 799 ($731 622) per QALY gained Cocaine use: Study 5: £450 471 ($698 549) per QALY gained Study 6: £197 623 ($306 455) per QALY gained Study 7: £967 573 ($1 500 422) per QALY gained Sensitivity analysis: web-based feedback could be cost-effective when cost was reduced to £1 ($1.55) from £15 ($23.26) Familia Unidas could be cost-effective if effect was sustained and intervention cost was £116 ($180) from £154 ($239)
White et al (2009),^31^ USA	Drug use (Opioid)	Individuals with private insurance (12–64 years old) (universal)	Intervention(s): (1) Theoretical misuse-deterrent/resistant prescription opioid (2) Existing extended-release prescription opioid (comparator)	CBA	Modelling (budget impact)	Perspective: third-party payer Time horizon: 1 year Reference year: 2006 Discount rate: none Currency: US$	Prescription drug costs, prescription opioid misuse dependence, or misuse-related healthcare costs, private payer costs	Potential cost-savings estimates from annual healthcare utilisation, cost from avoided misuse-related episode and prescription costs	Potential cost-savings to third-party payers from introducing a misuse-deterrent/resisters prescription opioid for the USA (assuming a privately insured cost structure) could range from approximately $0.6 ($0.748) billion to $1.6 ($2.0) billion per year, depending on different possible scenarios

ICER, incremental cost-effectiveness ratio; SFP:10-14, Strengthening Families Program 10–14; LST, Life Skills Training; Project ALERT, Adolescent, Learning, Experiences, Resistance and Training; CEA, cost-effectiveness analysis; WTP, willingness-to-pay; CUA, cost–utility analysis; QALY, quality-adjusted life-year; ISFP, Iowa Strengthening Families Program; CBA, cost–benefit analysis; RCT, randomised controlled trial; PYMU, past-year methamphetamine use case; SET, Social and Emotional Training; NPV, net present value; SBIRT, screening, brief intervention and referral to treatment; STRIVE, SupporT to Reunite, Involve and Value Each other.

### Main findings

#### Prevention of prescription opioid misuse

Four studies evaluated the cost-effectiveness of prescription opioid misuse prevention, three of which were conducted in the USA and one in South Korea. The interventions identified for the USA setting were a school-based programme, a family-centred programme and misuse-deterrent opioid formulations; a doctor's database assisting patient risk assessment was evaluated in the South Korean setting. All four studies utilised economic models to evaluate cost and benefit of intervention over 1- to 30-year time horizons.

Two of the studies evaluated misuse-deterrent opioid formulations against extended-release opioids. White et al^31^ conducted a CBA with a third-party payer perspective (i.e. private insurance), using a 1-year time horizon; Kumar et al^27^ conducted a CEA considering a health sector perspective with a 5-year time horizon. Both studies included direct costs related to healthcare utilisation.^27^ The White et al^31^ budget impact model estimated that there were potential cost-savings ranging from US$0.748 to US$2 billion for the insurance payer; however, the misuse-deterrent opioid prescription cost used in the study was a shadow cost or a similar cost of a branded opioid. In contrast, the misuse-deterrent opioid prescription costs used by Kumar et al^27^ were actual drug costs that resulted in significant costs to the healthcare system. Sensitivity analysis indicated that the model was sensitive to misuse-deterrent opioid price, where a 40% reduction in misuse-deterrent opioid prescription cost would result in cost neutrality.

The study by Crowley et al^21^ evaluated youth school-based programmes, a family-centred programme or a combination of both, and compared them with having no programme over a 6-year time horizon. Only intervention delivery costs were included in the analysis. A willingness-to-pay (WTP) threshold of US$8667 per case of non-medical opioid misuse prevented was established based on the average societal cost for youth engaged in nonmedical prescription opioid use. Three sets of interventions (Life Skills Training (LST) programme only, Strengthening Families Program 10–14 (SFP:10–14) + All Stars programme and SFP:10–14 + LST) were deemed to be cost-effective, given that the ICER fell below the predetermined WTP threshold of US$8667 to prevent one youth from misusing prescription opioids before the 12th grade.^21^

The South Korean study by Kim et al^25^ evaluated an opioid abuse preventive programme, ‘the Network System to Prevent Doctor-Shopping for Narcotics’, which allows doctors access to a database of a patient's previous narcotics use, allowing them to determine if a patient is at risk of misusing narcotics. Over a 30-year time horizon, compared with no programme, implementing the programme was determined to be cost-effective (US$227 per QALY; WTP threshold of US$31 362 per QALY) from a healthcare system perspective. Threshold analysis showed that the programme was 100% cost-effective even when using a WTP threshold of US$900 per QALY. Furthermore, including cost beyond the healthcare system under a societal perspective indicated that the intervention was cost-saving against having no programme.^25^

In summary, the literature showed that there are cost-effective preventive interventions for opioid misuse in the case of the school-based interventions (LST, SFP:10–14 + All Stars and SFP:10–14 + LST) and the doctor's database on assessing patient risk, as all fell under WTP thresholds.

#### Cannabis use prevention

Three studies evaluated cannabis use prevention interventions. Two studies evaluated school-based interventions and the third study evaluated two family-centred interventions and a web-based intervention.

A school-based educational programme integrated within different curriculums was evaluated by Mitchel et al^28^ in the USA. The CEA used a pre–post study design with a 10-month time frame. This study resulted in one of three integrated curriculums being effective. The study did not report an ICER, but instead presented an ‘effective cost’ or a cost-effectiveness ratio by dividing the societal cost of an intervention by the number of students with positive intervention effects. The study showed that the ‘effective cost’ for the religion curriculum with prevention education programme was US$181 per case prevented of cannabis and cigarette consumption.^28^ The second school-based intervention, Project ALERT (Adolescent, Learning, Experiences, Resistance and Training), was evaluated with an economic model in the Swedish setting by Deogan et al.^22^ Project ALERT aimed to prevent cannabis experimentation and continuation of use compared with a ‘do nothing’ scenario under a societal perspective. In their modelling, they included prevention of regular use of cannabis and transitioning to using other illicit drugs. The study found that the intervention was cost-effective at 20 years, with an ICER of US$2845 per QALY gained. When the time horizon was extended to a lifetime time horizon, the ICER reduced to US$1904 per QALY gained.^22^

The third cannabis prevention study by Pennington et al^30^ simulated three different targeted behavioural interventions identified from literature review. Two family-based interventions (Focus on Families and Familias Unidas) were evaluated against active comparators with short time horizons of 1 and 2 years, respectively. The cannabis model generated for the evaluation included intervention, crime and treatment of potential psychotic disorder costs that was described as a partial public sector perspective. Considering the reported WTP threshold of US$31 014, none of the interventions were cost-effective. Reported ICERs were US$153 915 305 per QALY gained (Focus on Families) and US$373 711 per QALY gained (Familias Unidas). The third intervention was modelled over 2 years and was a web-based intervention that resulted in an ICER of US$741 697 per QALY gained, which also exceeded the WTP threshold.

For cannabis use prevention, the school-based programme Project ALERT had promising cost-effectiveness evidence.

#### Illicit drug use prevention

Five studies were identified that looked at prevention of methamphetamine use, ecstasy use, cocaine use and general drug use. Evaluated preventive interventions were family-centred programmes; school-based interventions; screening, brief intervention and referral to treatment (SBIRT); and motivational interviewing. The first four were evaluated in a universal population whereas motivational interviewing was evaluated in an at-risk or indicated population.

Hajizadeh et al^24^ evaluated a family-centred intervention, ParentCorps, comparing it with a standard pre-kindergarten programme by using a model that simulated pre-kindergarten children over a lifetime. The societal perspective included healthcare (obesity, diabetes and drug use related), judiciary and productivity in addition to intervention costs. ParentCorps was shown to have a savings of US$4703 and an additional 0.27 QALYs.^24^ In the USA setting, an economic evaluation alongside an RCT evaluated the cost-effectiveness of a family-based intervention, Iowa Strengthening Families Program (ISFP), and a school-based intervention, LST, or a combination thereof, in preventing methamphetamine use. Guyll et al^23^ undertook economic analyses using both CEA and CBA. The perspective taken was from the employer and included productivity-related costs in addition to employer-funded healthcare costs. The CEA utilised a 5.5- to 6.5-year time horizon, whereas the modelled CBA evaluated a career-duration time horizon (from 18 to 65 years). ISFP, LST and a combination of LST and an adaptation of ISFP (SFP:10–14 + LST) were found to be effective and cost-effective under CBA. ICERs reported for CEA were US$31 664 (ISFP), US$6377 (LST) and US$78 206 (SFP:10–14 + LST) per past-year methamphetamine use case prevented. Modelled CBA results showed positive benefit–cost ratios of 3.84 (ISFP), 19.04 (LST) and 1.56 (SFP:10–14 + LST).^23^ Another school-based intervention, Social and Emotional Training (SET), was evaluated by Klapp et al in seventh to ninth grade students over 5 years. The study reports an intervention cost of US$596 per student and a benefit of US$8292 from avoided social burden of drug use, equivalent to US$13.9 per dollar spent.^26^ The final universal prevention study evaluated SBIRT compared with no SBIRT, under a healthcare perspective CBA for adults. The study reports annual cost-savings equal to a ratio of US$16 per dollars spent.^29^

Pennington et al^30^ assessed motivational interviewing for at-risk young gay and bisexual men, and a family-based intervention for newly homeless youth for cocaine use prevention. The modelling used a partial public sector perspective by including healthcare and criminal justice sector costs over a 2-year time horizon. Costs in the model included intervention, criminal justice and cocaine dependence treatment costs (including cocaine-specific hospital admission and cocaine-related cardiovascular treatment). Two motivational interviewing interventions were evaluated against educational videos in gay men. ICERs reported for the motivational interviewing interventions were US$698 549 and US$306 455 per QALY gained. The family intervention was evaluated against standard care by modelling a homeless youth population, and reported an ICER of US$1 500 422 per QALY gained.^30^

In the Pennington et al^30^ study, a targeted prevention strategy, motivational and cognitive behavioural intervention, was modelled for prevention of ecstasy use in people who occasionally use illict drugs. The intervention was modelled over a 1-year time horizon and included similar costs as mentioned previously. The intervention resulted in a ICER of US$731 622 per QALY gained.^30^

Several preventive interventions for illicit drug use are backed by value-for-money evidence. SBIRT, family-based interventions (ParentCorps and ISFP), school-based interventions (LST and SET) or a combination (SFP:10–14 + LST) had promising cost-effectiveness results.

### Quality assessment

Overall, most of the studies (73%) are of fair quality, except for two studies (18%) with good quality and one study (9%) with poor quality.^20^ Most of the studies satisfied the requirements for a good research question based on the Drummond criteria. Only 64% of studies reported the economic perspective taken by the evaluation. The evidence for intervention effectiveness was sourced from RCTs (18%), systematic reviews of multiple clinical studies (27%) and observational studies (64%). Hajizadeh et al^24^ utilised both a RCT and observational evidence to model cost-effectiveness. Costs and consequences identified were wide enough for the research question (82%) and covered the perspective taken (73%). Only 64% reported inclusion of capital and/or rollout costs. In terms of measurement of costs and consequences, 91% of the studies were judged to have adequate description and justification, and no studies omitted any identified costs and consequences. There was adequate reporting of valuation, with most studies using market values and all studies clearly identifying sources. Discounting was used in seven out of nine studies that reported time horizons longer than 1 year. Most studies (82%) conducted incremental cost and outcome analysis. The majority employed sensitivity analysis, which guided the conclusions (73%). Presentation of results were reported with the majority discussing generalisability (64%), comparison with existing evidence (73%), implementation (64%) and need for further research (82%). A summary of the quality assessment is presented in Table 2.

**Table 2:** Quality assessment

	Crowley et al (2014)^21^	Deogan et al (2015)^22^	Guyll et al (2011)^23^	Hajizadeh et al (2017)^24^	Kim et al (2021), South Korea^25^	Klapp et al (2017)^26^	Kumar et al (2019)^27^	Mitchel et al (1984)^28^	Paltzer et al (2019)^29^	Pennington et al (2018)^30^	White et al (2009)^31^
1. Was a well-defined question posed in answerable form?	No	Yes	Yes	Yes	Yes	Yes	Yes	Cannot tell	No	Yes	Yes
2. Was a comprehensive description of the competing alternatives given (i.e. can you tell who did what to whom, where and how often)?	Yes	Yes	Yes	Yes	Yes	Yes	No	No	Yes	No	No
3. Was the effectiveness of the programme or services established?	Yes	Yes	Yes	Yes	Yes	Yes	Yes	Yes	Yes	Yes	Yes
4. Were all the important and relevant costs and consequences for each alternative identified?	Cannot tell	Yes	Cannot tell	Yes	Yes	Yes	Cannot tell	No	Cannot tell	Cannot tell	Yes
5. Were costs and consequences measured accurately in appropriate physical units (e.g. hours of nursing time, number of physician visits, lost workdays, gained life-years)?	Cannot tell	Yes	Yes	Yes	Yes	Yes	Yes	Yes	Yes	Yes	Yes
6. Were the cost and consequences valued credibly?	Yes	Yes	Yes	Yes	Yes	Yes	Yes	Yes	Yes	Yes	Yes
7. Were costs and consequences adjusted for differential timing?	No	No	No	Yes	Yes	No	No	No	No	Yes	No
8. Was an incremental analysis of costs and consequences of alternatives performed?	Yes	Yes	Yes	No	Yes	Yes	Yes	No	Yes	Yes	Yes
9. Was allowance made for uncertainty in the estimates of costs and consequences?	No	Yes	Yes	Yes	Yes	No	Yes	No	Yes	Yes	Yes
10. Did the presentation and discussion of study results include all issues of concern to users?	Yes	Cannot tell	Cannot tell	Yes	Yes	Yes	Yes	No	Yes	Cannot tell	Yes
Yes	5	8	7	9	10	8	7	3	7	7	8
Cannot tell	3	1	1	1	0	2	2	6	2	1	2
No	2	1	2	0	0	0	1	1	1	2	0
Quality score	6	8.5	8	9	10	8	7.5	3.5	7.5	8	8
Judgement	Fair	Fair	Fair	Good	Good	Fair	Fair	Poor	Fair	Fair	Fair

## Discussion

### Summary of the main findings

This review is the first international review to comprehensively bring together economic evaluations of preventive interventions specifically for opioid misuse, cannabis and illicit drug use. The review distinguishes itself from other reviews that did not have a specific focus on cannabis use, opioid misuse and illicit drug use prevention.^15,16^ We found that there are preventive interventions that are supported by cost-effectiveness evidence that are of fair to good quality. In particular, school-based (LST, Project ALERT and SET) and family-centred programmes (ParentCorps and ISFP), or combinations of both (SFP:10–14 + All Stars, SFP:10–14 + LST), have demonstrated promising cost-effectiveness results across cannabis use, opioid misuse and illicit drug use prevention. Other preventive interventions with cost-effectiveness evidence are SBIRT for illicit drug use prevention and the Network System to Prevent Doctor-Shopping for Narcotics for problem opioid use prevention. Although multiple preventive interventions for cannabis use, opioid misuse and illicit drug use have been found to have positive effects, particularly in the short term,^11^ this review found that there are still limited numbers of full economic evaluations in this area. Most of the studies have been published in the past decade in the USA, with no economic evaluations found for low- and middle-income countries. Another review by Le et al^16^ also identified a similar lack of economic evaluations in low- and middle-income countries in their review of economic evaluations of mental health prevention and promotion.

Although the review intended to look into a broad range of preventive programmes, no studies were identified that focused on supply reduction, as all studies focused on demand reduction. Most programmes included in this review were universal and targeted young people (<18 years) through school settings. This is logical given that delaying initial consumption is typically the goal of cannabis use, opioid misuse and illicit drug use prevention. The majority of studies used economic modelling to simulate the cost-effectiveness of interventions. Economic models allow researchers to estimate population-level costs and effects over extended time periods. The models for substance use prevention projected costs and outcomes from 1-year to lifetime time horizons. Longer time horizons provided favourable cost-effectiveness results, from avoidance of longer-term costs to health and social systems. This trend is similar to alcohol and tobacco prevention interventions with lifetime time horizons included in the Nystrand et al^15^ review. A limitation of using a longer time horizon is that models use assumptions that may limit confidence in conclusions.

There were a variety of perspectives reported. The most common perspective was societal, which would be appropriate given the costs associated with substance use that fall outside the healthcare system, such as judiciary and productivity costs.^32^ This is consistent with the recommendation promoted by Neumann et al,^33^ as societal perspective reflects public interest. Some studies chose to evaluate narrow health sector or employer perspectives, most likely because of the difficulties of collecting costs for criminal justice and lost productivity associated with substance use. Using narrower perspectives are not necessarily inferior to a societal perspective, as choices regarding intervention or policy adoption depend on what level of evidence a decision maker requires. For instance, if an employer plans to implement a prevention programme, benefits of increased productivity may be enough for decision-making. This is the same when health payers use a healthcare perspective in deciding which programmes should be implemented.

All CBA studies reported cost-savings for the interventions evaluated, meaning the dollar value of the benefit gained was greater than every dollar being spent on the intervention. For CEA and CUA, Hajizadeh et al^24^ showed cost-savings and increased QALYs, which points toward ParentCorps intervention dominance. Interpretation of other ICERs would depend on a WTP threshold. Of the studies, four reported WTP thresholds, and three reported ICERs falling below the threshold designating the interventions as cost-effective.

### Methodological limitations of included studies

The Drummond checklist highlighted several limitations of the included studies. Multiple studies did not report perspectives that direct which resources should be identified, measured and valued. Several studies also evaluated interventions against active comparators, potentially minimising the incremental outcomes measured. In terms of evidence of effectiveness, most studies used observational data rather than RCTs, which may introduce bias. In addition, the economic models often relied on assumptions, especially when extrapolating a longer time horizon, adding to uncertainty in the results. Further research with economic evaluation alongside trials with long-term follow-up is required. Some studies did not address uncertainties in their results by failing to conduct sensitivity and uncertainty analysis, which could have increased confidence in the results. Although the Drummond checklist is clearly capable of identifying low-quality studies, it remains less effective in distinguishing high-quality studies.^20^

### Implications for policy and future directions for research

When considering which preventive interventions to roll out, decision makers should consider cost-effectiveness in addition to effectiveness data. Given the few studies noted in this review, further economic evaluations should be considered alongside intervention implementation in real-world settings, adding to the information available to decision makers. Of the interventions identified, ParentCorps for illicit drug use prevention and the Network System to Prevent Doctor-Shopping for Narcotics for problem opioid use prevention were of high quality and also highly cost-effective. The school-based interventions Project ALERT and SET were also cost-effective, but the evaluations were of fair quality.

The lack of economic evaluations in low- and middle-income countries is expected given that the Global Burden of Disease study revealed lower drug-attributable burden in lower-income countries. However, it is noteworthy that despite lower rates of illicit drug use in low- and middle-income countries, individuals with substance use problems have experienced higher mortality and have been less likely to receive treatment than those in high-income countries.^34,35^ Further research is required to establish cost-effectiveness evidence of preventive interventions for cannabis use, opioid misuse and illicit drug use in these contexts. The methodology used in previous alcohol prevention evaluations by Chisholm et al can be a basis for future research.^36,37^ This has the potential to bridge the gap in evidence to inform low- and middle-income countries regarding the impact of substance use prevention. In these studies, different alcohol prevention interventions were evaluated at different global contexts (e.g. world regions or low- and middle-income countries).

### Strengths and limitations of the review

The original review protocol reflected our intention for a large-scale review that included prevention of alcohol and tobacco use; however, the large number of included studies warranted separate reviews. Although this limits the scope of this review, it highlights the cost-effectiveness evidence specific to opioid misuse, cannabis use and illicit drug use prevention. Meta-analysis was not attempted because of the heterogeneity in design and methodology, a problem that is common to systematic reviews of economic evaluations.^38^ However, the review presented costs in a single reference year for comparison across studies. Another limitation of this review is that non-English studies were excluded, which may have resulted in the exclusion of potentially relevant articles in low- and middle-income countries where English is not the first language. There may also be other relevant articles that were unintentionally excluded from the use of limited online libraries and during the screening process. Given this limitation, grey literature search from cost-effectiveness registries was performed to minimise missed studies. Although no ROI studies were found, it was the intention of this review to include such studies as they may be more familiar to public sector decision makers. Most of the studies are from the past 15 years, and so are still relevant for consideration.

In conclusion, our review showed that there has been limited economic evaluation studies around opioid misuse, cannabis and illicit drug use prevention. The differences in context and methodologies of identified studies do not facilitate generalisability of the cost-effectiveness findings. The studies were of reasonable quality, indicating some promising cost-effectiveness evidence. In particular, ParentCorps, Project ALERT and SET, which were analysed from the societal perspective, and the Network System to Prevent Doctor-Shopping for Narcotics for problem opioid use prevention, which was analysed from the healthcare perspective, all showed cost-effective and were of high-quality evaluation. Further research is required to confirm the replication of the economic evaluation results, as well as to explore further context-specific piloting of promising programmes accompanied by economic evaluation.

## Supporting information

Faller et al. supplementary material Faller et al. supplementary material

## Data Availability

All data and materials are available within the manuscript and supplementary resources. Further information may be available by request to the corresponding author, J.F.
