# Supplementary material for: A systematic review of economic evaluations for opioid misuse, cannabis and illicit drug use prevention
Source: BJPsych Open. 2023 Aug 8;9(5):e149. doi: 10.1192/bjo.2023.515 (PMC10594094; doi:10.1192/bjo.2023.515)
Supplement: Faller et al. supplementary material [file S205647242300515Xsup001.docx]

Appendix 1: Search strategy (Embase)

| No. | Query | Results  (01/08/2019) | Results  (05/05/2021) |
| --- | --- | --- | --- |
| #1 | 'substance use'/exp | 468,870 | 528,583 |
| #2 | 'alcohol abuse'/exp | 36,642 | 42,102 |
| #3 | 'drug abuse'/exp | 109,851 | 124,284 |
| #4 | 'street drug'/de | 3,587 | 3,700 |
| #5 | ((alcohol OR drug* OR substance* OR 'ill* drug*' OR 'banned substance*' OR cannabi* OR marijuana OR cocaine OR heroin OR meth* OR 'street drug*') NEAR/2 (use OR misuse OR abuse OR intake OR consum*)):ti | 85,827 | 96,795 |
| #6 | ((alcohol OR drug* OR substance* OR 'ill* drug*' OR 'banned substance*' OR cannabi* OR marijuana OR cocaine OR heroin OR meth* OR 'street drug*') NEAR/2 (use OR misuse OR abuse OR intake OR consum*)):ab | 333,755 | 382,032 |
| #7 | #1 OR #2 OR #3 OR #4 OR #5 OR #6 | 775,862 | 876,252 |
| #8 | prevent*:ti OR promot*:ti OR treat*:ti OR manag*:ti | 2,783,298 | 1,207,789 |
| #9 | prevent*:ab OR promot*:ab OR treat*:ab OR manag*:ab | 8,755,720 | 4,386,303 |
| #10 | #8 OR #9 | 9,844,974 | 4,922,369 |
| #11 | #7 AND #10 | 307,913 | 182,321 |
| #12 | 'cost benefit analysis'/de | 81,166 | 86,660 |
| #13 | 'cost benefit analysis':ti | 1,693 | 1,791 |
| #14 | 'cost benefit analysis':ab | 3,589 | 3,928 |
| #15 | 'economic evaluation'/exp | 290,692 | 317,786 |
| #16 | 'economic evaluation*':ti | 6,400 | 7,297 |
| #17 | 'economic evaluation*':ab | 11,654 | 13,757 |
| #18 | 'cost analys?s':ti | 3,907 | 4,463 |
| #19 | 'cost analys?s':ab | 7,668 | 8,831 |
| #20 | 'return on investment':ti | 404 | 467 |
| #21 | 'return on investment':ab | 1,942 | 2,354 |
| #22 | 'return to investment':ti | 1 | 1 |
| #23 | 'return to investment':ab | 8 | 9 |
| #24 | 'cost effectiveness analysis'/de | 142,752 | 158,570 |
| #25 | 'cost effectiveness analysis':ti | 6,786 | 7,986 |
| #26 | 'cost effectiveness analysis':ab | 8,653 | 10,014 |
| #27 | 'cost utility analysis'/de | 9,006 | 10,297 |
| #28 | 'cost utility analysis':ti | 1,491 | 1,714 |
| #29 | 'cost utility analysis':ab | 2,476 | 2,858 |
| #30 | #12 OR #13 OR #14 OR #15 OR #16 OR #17 OR #18 OR #19 OR #20 OR #21 OR #22 OR #23 OR #24 OR #25 OR #26 OR #27 OR #28 OR #29 | 298,098 | 326,149 |
| #31 | #11 AND #30 | 5,574 | 3,739 |
| #32 | #11 AND #30 AND [humans]/lim AND [english]/lim AND [embase]/lim | 4,266 | 2,880 |
| #33 | #11 AND #30 AND ([article]/lim OR [article in press]/lim OR [erratum]/lim) AND [humans]/lim AND [english]/lim AND [embase]/lim | 2,068 | 1,321 |
|  |  | 2,187 | |

Note: The highlighted term ‘treat*’ was not included in the 05/05/2021 to reflect the preventive focus of this review.

Search strategy (Medline)

| # | Query | Results  (01/08/2019) | Results  (05/05/2021) |
| --- | --- | --- | --- |
| S1 | (MH "Marijuana Use") OR (MH "Marijuana Abuse") OR (MH "Inhalant Abuse") OR (MH "Cocaine-Related Disorders") OR (MH "Amphetamine-Related Disorders") OR (MH "Alcoholism") OR (MH "Binge Drinking") OR (MH "Alcoholic Intoxication") OR (MH "Substance Abuse, Intravenous") OR (MH "Substance Abuse, Oral") OR (MH "Tobacco Use Disorder") OR (MH "Phencyclidine Abuse") OR (MH "Drug Overdose") | 132,849 | 141,868 |
| S2 | (MH "Alcohol Drinking+") | 65,396 | 71,123 |
| S3 | (MH "Crack Cocaine") | 1,378 | 1,441 |
| S4 | TI (alcohol OR drug* OR substance* OR "ill* drug*" OR "banned substance*" OR cannabi* OR marijuana OR cocaine OR heroin OR meth* OR "street drug*") N2 (use or misuse or abuse or intake or consum*) | 75,784 | 87,598 |
| S5 | AB (alcohol OR drug* OR substance* OR "ill* drug*" OR "banned substance*" OR cannabi* OR marijuana OR cocaine OR heroin OR meth* OR "street drug*") N2 (use or misuse or abuse or intake or consum*) | 293,536 | 332,602 |
| S6 | S1 OR S2 OR S3 OR S4 OR S5 | 428,580 | 476,835 |
| S7 | TI prevent* or promot* or treat* or manag* | 7,418,339 | 3,149,512 |
| S8 | AB prevent* or promot* or treat* or manag* | 7,817,921 | 3,940,864 |
| S9 | S7 OR S8 | 7,927,888 | 4,092,459 |
| S10 | S6 AND S9 | 176,339 | 99,231 |
| S11 | (MH "Cost-Benefit Analysis") | 76,614 | 84,003 |
| S12 | TI "Cost Benefit Analys?s" | 1,323 | 1,444 |
| S13 | AB "Cost Benefit Analys?s" | 3,213 | 3,589 |
| S14 | TI "economic evaluation*" | 4,747 | 5,339 |
| S15 | AB "economic evaluation*" | 8,199 | 9,588 |
| S16 | (MH "Costs and Cost Analysis") | 47,259 | 49,429 |
| S17 | TI "Cost Analys?s" | 2,620 | 2,980 |
| S18 | AB "Cost Analys?s" | 4,771 | 5,473 |
| S19 | TI "return on investment" | 331 | 402 |
| S20 | AB "return on investment" | 1,482 | 1,801 |
| S21 | TI "return to investment" | 1 | 1 |
| S22 | AB "return to investment" | 5 | 5 |
| S23 | TI "cost effectiveness analys?s" | 4,666 | 5,421 |
| S24 | AB "cost effectiveness analys?s" | 7,607 | 8,718 |
| S25 | TI "cost utility analys?s" | 982 | 1,128 |
| S26 | AB "cost utility analys?s" | 2,152 | 2,549 |
| S27 | S11 OR S12 OR S13 OR S14 OR S15 OR S16 OR S17 OR S18 OR S19 OR S20 OR S21 OR S22 OR S23 OR S24 OR S25 OR S26 | 134,861 | 146,378 |
| S28 | S10 AND S27 | 2,236 | 1,378 |
| S29 | S10 AND S27 (Limiters – English Language; Human; Publication Type: Introductory Journal Article, Journal Article Search modes - Boolean/Phrase) | 1,888 | 1,184 |
|  |  | 2,017 | |

Note: The highlighted term ‘treat*’ was not included in the 05/05/2021 to reflect the preventive focus of this review.

Search strategy (Cinahl)

| # | Query | Results  (01/08/2019) | Results  (05/05/2021) |
| --- | --- | --- | --- |
| S1 | (MH "Substance Abuse+") | 60,314 | 70,418 |
| S2 | (MH "Substance Abusers+") | 7,485 | 8,943 |
| S3 | (MH "Alcoholism") OR (MH "Alcohol Drinking+") | 39,401 | 46,632 |
| S4 | (MH "Street Drugs+") | 5,313 | 6,091 |
| S5 | TI (alcohol OR drug* OR substance* OR "ill* drug*" OR "banned substance*" OR cannabi* OR marijuana OR cocaine OR heroin OR meth* OR "street drug*") N2 (use or misuse or abuse or intake or consum*) | 37,618 | 45,077 |
| S6 | AB (alcohol OR drug* OR substance* OR "ill* drug*" OR "banned substance*" OR cannabi* OR marijuana OR cocaine OR heroin OR meth* OR "street drug*") N2 (use or misuse or abuse or intake or consum*) | 91,085 | 116,201 |
| S7 | S1 OR S2 OR S3 OR S4 OR S5 OR S6 | 152,277 | 184,780 |
| S8 | TI prevent* OR promot* OR treat* OR manag* | 1,637,100 | 978,416 |
| S9 | AB prevent* OR promot* OR treat* OR manag* | 1,692,174 | 1,125,112 |
| S10 | S8 OR S9 | 1,737,401 | 1,184,751 |
| S11 | S7 AND S10 | 63,123 | 43,782 |
| S12 | (MH "Cost Benefit Analysis") | 29,391 | 35,296 |
| S13 | TI "Cost Benefit Analys?s" | 335 | 402 |
| S14 | AB "Cost Benefit Analys?s" | 754 | 945 |
| S15 | TI "economic evaluation*" | 2,141 | 2,507 |
| S16 | AB "economic evaluation*" | 3,004 | 3,776 |
| S17 | (MH "Costs and Cost Analysis") | 16,078 | 18,609 |
| S18 | TI "Cost Analys?s" | 1,011 | 1,211 |
| S19 | AB "Cost Analys?s" | 1,351 | 1,680 |
| S20 | TI "return on investment" | 276 | 319 |
| S21 | AB "return on investment" | 732 | 960 |
| S22 | TI "return to investment" | 276 | 319 |
| S23 | AB "return to investment" | 732 | 960 |
| S24 | TI "cost effectiveness analys?s" | 2,162 | 2,661 |
| S25 | AB "cost effectiveness analys?s" | 2,516 | 3,195 |
| S26 | TI "cost utility analys?s" | 489 | 589 |
| S27 | AB "cost utility analys?s" | 723 | 919 |
| S28 | S12 OR S13 OR S14 OR S15 OR S16 OR S17 OR S18 OR S19 OR S20 OR S21 OR S22 OR S23 OR S24 OR S25 OR S26 OR S27 | 49,389 | 58,755 |
| S29 | S11 AND S28 | 896 | 596 |
| S30 | S11 AND S28 (Limiters – English Language; Peer Reviewed; Human; Publication Type: Corrected Article, Journal Article) | 516 | 293 |
|  |  | 545 | |

Note: The highlighted term ‘treat*’ was not included in the 05/05/2021 to reflect the preventive focus of this review.

Search strategy (PsychInfo)

| # | Query | Results  (01/08/2019) | Results  (05/05/2021) |
| --- | --- | --- | --- |
| S1 | DE "Marijuana Usage" OR DE "Substance Use Disorder" OR DE "Addiction" OR DE "Alcohol Use Disorder" OR DE "Cannabis Use Disorder" OR DE "Drug Abuse" OR DE "Inhalant Abuse" OR DE "Opioid Use Disorder" OR DE "Tobacco Use Disorder" | 64,665 | 71,594 |
| S2 | DE "Alcohol Abuse" OR DE "Alcoholism" OR DE "Binge Drinking" OR DE "Alcohol Drinking Attitudes" OR DE "Alcohol Drinking Patterns" OR DE "Social Drinking" OR DE "Underage Drinking" OR DE "Alcohol Use Disorder" OR DE "Alcohol Abuse" OR DE "Alcohol Intoxication" | 73,544 | 78,203 |
| S3 | DE "Drug Abuse" OR DE "Inhalant Abuse" OR DE "Polydrug Abuse" OR DE "Drug Addiction" | 56,349 | 58,817 |
| S4 | TI (alcohol OR drug* OR substance* OR "ill* drug*" OR "banned substance*" OR cannabi* OR marijuana OR cocaine OR heroin OR meth* OR "street drug*") N2 (use or misuse or abuse or intake or consum*) | 52,612 | 58,367 |
| S5 | AB (alcohol OR drug* OR substance* OR "ill* drug*" OR "banned substance*" OR cannabi* OR marijuana OR cocaine OR heroin OR meth* OR "street drug*") N2 (use or misuse or abuse or intake or consum*) | 169,119 | 186,124 |
| S6 | S1 OR S2 OR S3 OR S4 OR S5 | 227,072 | 246,931 |
| S7 | TI prevent* OR promot* OR treat* OR manag* | 1,433,999 | 719,734 |
| S8 | AB prevent* OR promot* OR treat* OR manag* | 1,506,833 | 852,829 |
| S9 | S7 OR S8 | 1,510,452 | 859,085 |
| S10 | S6 AND S9 | 124,189 | 59,776 |
| S11 | TI "Cost Benefit Analys?s" | 190 | 201 |
| S12 | AB "Cost Benefit Analys?s" | 956 | 1,036 |
| S13 | TI "economic evaluation*" | 529 | 614 |
| S14 | AB "economic evaluation*" | 1,359 | 1,602 |
| S15 | DE "Costs and Cost Analysis" | 17,287 | 18,731 |
| S16 | TI "Cost Analys?s" | 192 | 208 |
| S17 | AB "Cost Analys?s" | 633 | 683 |
| S18 | TI "return on investment" | 109 | 129 |
| S19 | AB "return on investment" | 773 | 871 |
| S20 | TI "return to investment" | 109 | 129 |
| S21 | AB "return to investment" | 773 | 871 |
| S22 | TI "cost effectiveness analys?s" | 383 | 427 |
| S23 | AB "cost effectiveness analys?s" | 1,022 | 1,156 |
| S24 | TI "cost utility analys?s" | 75 | 85 |
| S25 | AB "cost utility analys?s" | 304 | 361 |
| S26 | S11 OR S12 OR S13 OR S14 OR S15 OR S16 OR S17 OR S18 OR S19 OR S20 OR S21 OR S22 OR S23 OR S24 OR S25 | 20,202 | 22,115 |
| S27 | S10 AND S26 | 1,092 | 554 |
| S28 | S10 AND S26 (Limiters – Peer Reviewed; Publication Type: Peer Reviewed Journal; English; Population Group: Human; Document Type: Erratum/Correction, Journal Article, Retraction) | 835 | 426 |
|  |  | 870 | |

Note: The highlighted term ‘treat*’ was not included in the 05/05/2021 to reflect the preventive focus of this review.

Search strategy (EconLit)

| # | Query | Results  (01/08/2019) | Results  (05/05/2021) |
| --- | --- | --- | --- |
| S1 | (ZW "substance use disorders") | 2 | 2 |
| S2 | (ZW "alcohol") or (ZW "alcohol availability, health, alcohol consumption") or (ZW "alcohol consumption") or (ZW "alcohol consumption, alcohol tax, binge drinking, beer, wine and spirits") or (ZW "alcohol misuse, morbidity, ordered probit, tobit, eq5d") or (ZW "alcohol, demand model, patterns of consumption") or (ZW "alcohol.") or (ZW "alcoholism") | 49 | 51 |
| S3 | (ZW "drug addiction") or (ZW "drug traffic") or (ZW "drugs") or (ZW "drugs of abuse") | 1,438 | 1,595 |
| S4 | TI (alcohol OR drug* OR substance* OR "ill* drug*" OR "banned substance*" OR cannabi* OR marijuana OR cocaine OR heroin OR meth* OR "street drug*") N2 (use or misuse or abuse or intake or consum*) | 1,021 | 1,084 |
| S5 | AB (alcohol OR drug* OR substance* OR "ill* drug*" OR "banned substance*" OR cannabi* OR marijuana OR cocaine OR heroin OR meth* OR "street drug*") N2 (use or misuse or abuse or intake or consum*) | 6,792 | 7,775 |
| S6 | S1 OR S2 OR S3 OR S4 OR S5 | 8,565 | 9,709 |
| S7 | TI prevent* or promot* or treat* or manag* | 341,062 | 333,678 |
| S8 | AB prevent* or promot* or treat* or manag* | 349,052 | 343,295 |
| S9 | S7 OR S8 | 349,822 | 344,159 |
| S10 | S6 AND S9 | 2,607 | 2,245 |
| S11 | (ZW "cost benefit") or (ZW "cost benefit analysis") | 887 | 1,001 |
| S12 | TI "Cost Benefit Analys?s" | 978 | 1,019 |
| S13 | AB "Cost Benefit Analys?s" | 1,751 | 1,885 |
| S14 | (ZW "economic evaluation") | 2 | 2 |
| S15 | TI "economic evaluation*" | 761 | 810 |
| S16 | AB "economic evaluation*" | 756 | 845 |
| S17 | (ZW "cost analysis") | 1 | 1 |
| S18 | TI "cost analys?s" | 590 | 627 |
| S19 | AB "cost analys?s" | 756 | 824 |
| S20 | (ZW "return on investment") | 3 | 9 |
| S21 | TI "return on investment" | 121 | 128 |
| S22 | AB "return on investment" | 599 | 682 |
| S23 | TI "return to investment" | 121 | 128 |
| S24 | AB "return to investment" | 599 | 682 |
| S25 | (ZW "cost effective") or (ZW "cost effectiveness") or (ZW "cost effectiveness analysis") | 527 | 623 |
| S26 | TI "cost effectiveness analys?s" | 307 | 328 |
| S27 | AB "cost effectiveness analys?s" | 412 | 453 |
| S28 | TI "cost utility analys?s" | 52 | 60 |
| S29 | AB "cost utility analys?s" | 98 | 118 |
| S30 | S11 OR S12 OR S13 OR S14 OR S15 OR S16 OR S17 OR S18 OR S19 OR S20 OR S21 OR S22 OR S23 OR S24 OR S25 OR S26 OR S27 OR S28 OR S29 | 6,460 | 7,002 |
| S31 | S10 AND S30 | 62 | 42 |
| S32 | S10 AND S30 (Limiters - Publication Type: Journal Article Search modes - Boolean/Phrase) | 50 | 34 |
|  |  | 55 | |

Note: The highlighted term ‘treat*’ was not included in the 05/05/2021 to reflect the preventive focus of this review.

Appendix 2: Quality assessment

|  | | **Deogan et al. (2015)** | **Guyll et al. (2011)** | **Hajizadeh et al. (2017)** | **Kim et al. (2021), South Korea** | **Klapp et al. (2017)** | **Kumar et al. (2019)** | **MaxCrowley et al. (2014)** | **Mitchel et al. (1984)** | **Paltzer et al. (2019)** | **Pennington et al. (2018)** | **White et al. (2009)** |
| --- | --- | --- | --- | --- | --- | --- | --- | --- | --- | --- | --- | --- |
|  | **1 Was a well-defined question posed in answerable form?** | | | | | | | | | | | |
| 1.1 Did the study examine both costs and effects of the service(s) or programme(s) over an appropriate time horizon? | | Yes | Yes | Yes | Yes | Yes | Yes | Yes | Can't tell | Yes | Yes | Yes |
| 1.2 Did the study involve a comparison of alternatives? | | Yes | Yes | Yes | Yes | Yes | Yes | Yes | Yes | Yes | Yes | Yes |
| 1.3 Was a perspective for the analysis stated and was the study placed in any particular decision-making context? | | Yes | Yes | Yes | Yes | Yes | Yes | No | No | No | Yes | Yes |
| 1.4 Were the patient population and any relevant subgroups adequately defined? | | Yes | Yes | Yes | Yes | Yes | Yes | Yes | Yes | Yes | Yes | Yes |
|  | **2 Was a comprehensive description of the competing alternatives given? (i.e. can you tell who did what to whom, where, and how often?)** | | | | | | | | | | | |
| 2.1 Were any relevant alternatives omitted? | | No | No | No | No | No | No | No | Yes | No | No | Yes |
| 2.2 Was (should) a ‘do nothing’ alternative (be) considered? | | Yes | Yes | Yes | Yes | Yes | No | Yes | Yes | Yes | No | No |
| 2.3 Were relevant alternatives identified for the patient subgroups? | | Can't tell | Can't tell | Can't tell | Can't tell | Can't tell | Can't tell | Can't tell | Can't tell | Can't tell | Can't tell | Can't tell |
|  | **3 Was the effectiveness of the programmes or services established?** | | | | | | | | | | | |
| 3.1 Was this done through a randomized controlled clinical trial? If so, did the trial protocol reflect what would happen in regular practice? | | No | Yes | No | No | No | No | Yes | No | No | No | No |
| 3.2 Were effectiveness data collected and summarized through a systematic overview of clinical studies? If so, were the search strategy and rules for inclusion or exclusion outlined? | | Yes | No | Yes | No | No | No | No | No | No | Yes | No |
| 3.3 Were observational data or assumptions used to establish effectiveness? If so, were any potential biases recognized? | | No | No | Yes | Yes | Yes | Yes | No | Yes | Yes | No | Yes |
|  | **4 Were all the important and relevant costs and consequences for each alternative identified?** | | | | | | | | | | | |
| 4.1 Was the range wide enough for the research question at hand? | | Yes | Yes | Yes | Yes | Yes | Yes | Can't tell | No | Yes | Yes | Yes |
| 4.2 Did it cover all relevant perspectives? (Possible perspectives include those of patients and third-party payers; other perspectives may also be relevant depending on the particular analysis.) | | Yes | Yes | Yes | Yes | Yes | Yes | Can't tell | No | No | Yes | Yes |
| 4.3 Were capital costs, as well as operating costs, included? | | Yes | Can't tell | Yes | Yes | Yes | Can't tell | Yes | Yes | Can't tell | Can't tell | Yes |
|  | **5 Were costs and consequences measured accurately in appropriate physical units prior to valuation (e.g. hours of nursing time, number of physician visits, lost work-days, gained life-years)?** | | | | | | | | | | | |
| 5.1 Were the sources of resource utilization described and justified? | | Yes | Yes | Yes | Yes | Yes | Yes | Can't tell | Yes | Yes | Yes | Yes |
| 5.2 Were any of the identified items omitted from measurement? If so, does this mean that they carried no weight in the subsequent analysis? | | No | No | No | No | No | No | No | No | No | No | No |
| 5.3 Were there any special circumstances (e.g. joint use of resources) that made measurement difficult? Were these circumstances handled appropriately? | | Can't tell | No | No | Can't tell | No | No | Can't tell | Can't tell | No | No | No |
|  | **6 Were costs and consequences valued credibly?** | | | | | | | | | | | |
| 6.1 Were the sources of all values clearly identified? (Possible sources include market values, patient or client preferences and views, policymakers’ views, and health professionals’ judgements.) | | Yes | Yes | Yes | Yes | Yes | Yes | Yes | Yes | Yes | Yes | Yes |
| 6.2 Were market values employed for changes involving resources gained or depleted? | | Yes | Yes | Yes | Yes | Yes | Yes | Yes | No | Yes | Yes | Yes |
| 6.3 Where market values were absent (e.g. volunteer labour), or market values did not reflect actual values (e.g. clinic space donated at a reduced rate), were adjustments made to approximate market values? | | Can't tell | Can't tell | Can't tell | Can't tell | Can't tell | Can't tell | Can't tell | Can't tell | Can't tell | Can't tell | Can't tell |
| 6.4 Was the valuation of consequences appropriate for the question posed (i.e. has the appropriate type or types of analysis—cost-effectiveness, cost–benefit—been selected)? | | Yes | Yes | Yes | Yes | Yes | Yes | Yes | Yes | Yes | Yes | Yes |
|  | **7 Were costs and consequences adjusted for differential timing?** | | | | | | | | | | | |
| 7.1 Were costs and consequences that occur in the future ‘discounted’ to their present values? | | Yes | Yes | Yes | Yes | Yes | No | Yes | Yes | No | Yes | No |
| 7.2 Was any justification given for the discount rate(s) used? | | No | No | Yes | Yes | No | No | No | No | No | Yes | No |
|  | **8 Was an incremental analysis of costs and consequences of alternatives performed?** | | | | | | | | | | | |
| 8.1 Were the additional (incremental) costs generated by one alternative over another compared to the additional effects, benefits, or utilities generated? | | Yes | Yes | No | Yes | Yes | Yes | Yes | No | Yes | Yes | Yes |
|  | **9 Was uncertainty in the estimates of costs and consequences adequately characterized?** | | | | | | | | | | | |
| 9.1 If patient-level data on costs or consequences were available, were appropriate statistical analyses performed? | | Can't tell | Can't tell | Can't tell | Yes | Can't tell | Can't tell | Can't tell | Yes | Yes | Can't tell | Can't tell |
| 9.2 If a sensitivity analysis was employed, was justification provided for the form(s) of sensitivity analysis employed and the ranges or distributions of values (for key study parameters)? | | Yes | Yes | Yes | Yes | No | Yes | No | No | Yes | Yes | Yes |
| 9.3 Were the conclusions of the study sensitive to the uncertainty in the results, as quantified by the statistical and/or sensitivity analysis? | | Yes | Yes | Yes | Yes | Yes | Yes | Yes | No | Yes | Yes | Yes |
| 9.4 Was heterogeneity in the patient population recognized, for example by presenting study results for relevant subgroups? | | Yes | No | No | No | Yes | Can't tell | No | Yes | No | No | Yes |
|  | **10 Did the presentation and discussion of study results include all issues of concern to users?** | | | | | | | | | | | |
| 10.1 Were the conclusions of the analysis based on some overall index or ratio of costs to consequences (e.g. cost-effectiveness ratio)? If so, was the index interpreted intelligently or in a mechanistic fashion? | | Yes | Yes | Yes | Yes | Yes | Yes | Yes | No | Yes | Yes | Yes |
| 10.2 Were the results compared with those of others who have investigated the same question? If so, were allowances made for potential differences in study methodology? | | Yes | No | Yes | Yes | Yes | Yes | Yes | No | Yes | Can't tell | Yes |
| 10.3 Did the study discuss the generalizability of the results to other settings and patient/client groups? | | No | Yes | Yes | Yes | Yes | No | Yes | No | Yes | No | Yes |
| 10.4 Did the study allude to, or take account of, other important factors in the choice or decision under consideration (e.g. distribution of costs and consequences, or relevant ethical issues)? | | Yes | Yes | Yes | Yes | Yes | Yes | Yes | No | Yes | Yes | Yes |
| 10.5 Did the study discuss issues of implementation, such as the feasibility of adopting the ‘preferred’ programme given existing financial or other constraints, and whether any freed resources could be redeployed to other worthwhile programmes? | | No | No | Yes | Yes | Yes | Yes | Yes | No | Yes | No | Yes |
| 10.6 Were the implications of uncertainty for decision-making, including the need for future research, explored? | | Yes | No | Yes | Yes | Yes | Yes | Yes | No | Yes | Yes | Yes |
| Judgement | | FAIR | FAIR | GOOD | GOOD | FAIR | FAIR | FAIR | POOR | FAIR | FAIR | FAIR |
